# Deterministic versus stochastic model of reprogramming: new evidence from cellular barcoding technique

**DOI:** 10.1098/rsob.160311

**Published:** 2017-04-26

**Authors:** Anastasia M. Yunusova, Veniamin S. Fishman, Gennady V. Vasiliev, Nariman R. Battulin

**Affiliations:** 1 Laboratory of Developmental Genetics, Institute of Cytology and Genetics, Novosibirsk 630090, Russia; 2 Novosibirsk State University, Novosibirsk 630090, Russia; 3 Sector of Genomic Investigation, Institute of Cytology and Genetics, Novosibirsk 630090, Russia

**Keywords:** induced pluripotent stem cells, reprogramming to pluripotency, cellular barcoding, cell fate decisions

## Abstract

Factor-mediated reprogramming of somatic cells towards pluripotency is a low-efficiency process during which only small subsets of cells are successfully reprogrammed. Previous analyses of the determinants of the reprogramming potential are based on average measurements across a large population of cells or on monitoring a relatively small number of single cells with live imaging. Here, we applied lentiviral genetic barcoding, a powerful tool enabling the identification of familiar relationships in thousands of cells. High-throughput sequencing of barcodes from successfully reprogrammed cells revealed a significant number of barcodes from related cells. We developed a computer model, according to which a probability of synchronous reprogramming of sister cells equals 10–30%. We conclude that the reprogramming success is pre-established in some particular cells and, being a heritable trait, can be maintained through cell division. Thus, reprogramming progresses in a deterministic manner, at least at the level of cell lineages.

## 1. Introduction

Direct reprogramming of somatic cells to a pluripotent state can be achieved by overexpression of Oct4, Sox2, Klf4 and c-Myc (OSKM) transcription factors [1,2]. Reprogramming is accompanied by resetting the epigenome of somatic cells, yielding induced pluripotent stem cells (iPSCs) that functionally and molecularly resemble embryonic stem cells derived from early embryos [3–6]. However, reprogramming with Yamanaka's typical recipe (OSKM cocktail) is an extremely inefficient process—only small subsets of the heterogeneous cellular population are able to generate pluripotent progeny. Monitoring the progression of cells through the reprogramming process revealed that almost all cells transit into the initiation phase with the downregulation of somatic cell genes followed by the activation of early pluripotency markers [7]. By contrast, only rare somatic cells pass through a second wave where the core pluripotency network is stably maintained [8]. The difference between these ‘lucky’ minority and cells that are refractory to reprogramming remains elusive.

Different models have been proposed to explain this phenomenon. A stochastic model, supporter of chaotic, uncoordinated changes, posits that all cells are equally likely to reprogram at any given cell division but reprogramming events may or may not be achieved (random event). Therefore, iPSCs appear at different random times, and it is not possible to predict whether or when the progenies of somatic cells become an iPSC. According to the other deterministic model, particular ‘elite’ cells within the populations are predisposed to reprogramming and iPSCs appear at a fixed time as reprogramming events in these cells would be synchronized [9]. A strong support for the stochastic model comes from the clonal analysis of single B cells, demonstrating that almost all donor cells eventually give rise to iPSCs on continued growth and reprogramming factor expression [10]. Moreover, single-cell expression analysis of early reprogramming did not reveal a specific sequential order of gene activation between cells, thus proving the stochastic model at least in the early phase of reprogramming [11].

Nevertheless, evidence for the deterministic manner of reprogramming is also supported by experimental findings. For instance, Guo *et al.* [12] identified a privileged subset of fast-cycling bone marrow cells that is highly efficient in reprogramming. Another interesting example is Muse (multilineage-differentiating stress-enduring) cells in human fibroblasts that selectively become iPSCs, unlike the majority of cells that remain refractory to reprogramming [13]. It should be noted that the ‘privileged state’ could be achieved by transient overexpression of C/EBPα together with OSKM transduction [14], or depletion of Mbd3/NurD, the predominant molecular block that prevents the deterministic trajectory of induced pluripotency [15]. Taken together, these findings challenge previous assumptions about the stochastic nature of reprogramming [16].

To investigate this important matter in more detail (on a cell lineages resolution), we used the cellular barcoding method for the simultaneous tracking of progenies of thousands of cells during the reprogramming process. Analysis of barcodes from successfully reprogrammed cells revealed that individual daughters that originated from the same progenitor cell predominantly share the same reprogramming fate: if one daughter cell contributes to a lineage that gives rise to pluripotent cells, its paired sibling also does so. We suppose that the potential of reprogramming is predetermined and inherited during cell division.

## 2. Results

As a starting cell population, we chose OG2 mouse embryonic fibroblasts (MEFs) stably carrying an *Oct4* promoter-driven GFP reporter, thereby assisting in reprogramming tracking [17]. The Yamanaka factors were introduced by a single doxycycline (DOX) inducible polycistronic lentivirus; thus, the factor expression could be initiated whenever required by adding DOX to the culture medium. Besides, cells were also transduced with lentivirus encoding M2 reverse tetracycline transactivator (M2rtTA) that drives reprogramming factors expression in the presence of DOX [18]. It is important to note that M2rtTA lentiviruses also contain a variable random sequence tag or DNA ‘barcode’, the main protagonist of our study. On integration, a barcode introduces a unique and inheritable mark into the genome, allowing the clonal progenies to be tracked over time [19]. Thus, progenies descending from one labelled cell share the same barcode and could be easily identified by high-throughput sequencing. It is worth noting that the starting cell population will have random integrations of lentiviral vectors and, consequently, different expression levels of reprogramming factors. However, for our study, this is unlikely to have any major impact because we analyse clonally related sister cells, which originate from a common progenitor and therefore have a same viral integrations.

Our reprogramming experiments were terminated after one week of the reprogramming timeline; thus we focused on cells with rapid response to reprogramming factors expression.

**Figure 1.**
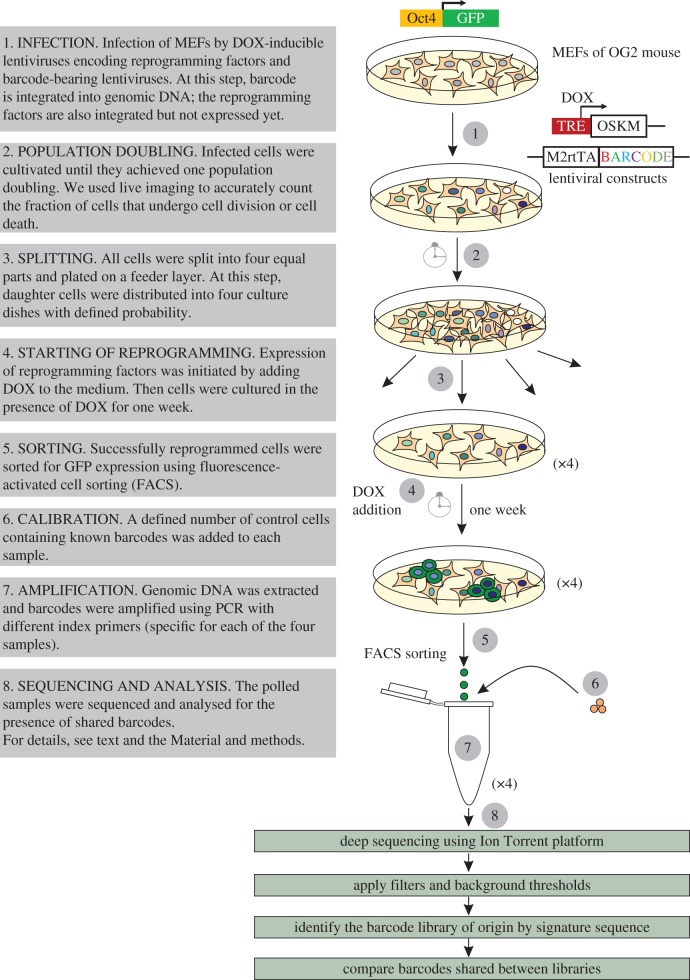
Experimental workflow.

To determine whether the reprogramming potential is symmetric between sister cells, we devised the following experimental strategy (figure 1). First, we transduced a known number of MEFs with the aforementioned combination of lentiviruses and allowed them to divide several times before factor induction. Then cells were split into four culture dishes, thus daughters of the same cell were represented by different dishes with high probability (e.g. 75% for any two related cells). Only after splitting, we started reprograming by adding DOX to each culture dish. After one week, we sorted successfully reprogrammed cells for GFP and recovered their barcodes using PCR and high-throughput sequencing. Comparing shared and distinct barcodes between different dishes, we could establish how many sister cells were synchronously reprogrammed. If the potential of reprogramming is largely predetermined, the fraction of shared barcodes will be significantly higher than accidental, assuming that the potential is inherited, and each sister cell will generate pluripotent progenies over a short experimental timeline (one week). In a stochastic model, among the barcodes of successfully reprogrammed cells, we will observe barcodes of unrelated cells that reprogrammed accidentally, just by random chance.

### 2.1. Clonally related cells share the same reprogramming fate

**Figure 2.**
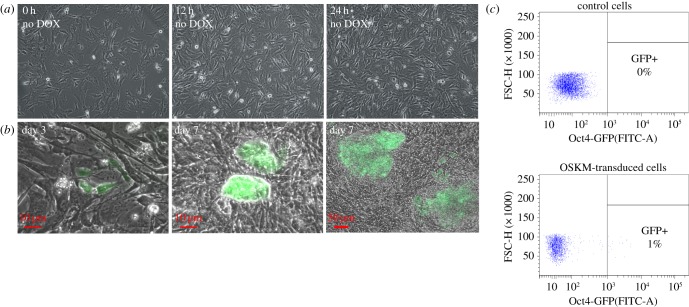
OG2-MEFs are rapidly converted to Oct4-GFP-positive cells after DOX induction. (*a*) Time-lapse live images of OG2 MEFs transduced with OSKM and cultured without DOX at indicated time point. (*b*) Generation of Oct4-GFP-positive cells: first GFP-positive cells appeared on day 3 of culturing in mouse iPSC medium supplemented with AGi and DOX; by day 7 they formed GFP-positive colonies of different sizes. (*c*) Fluorescence-activated cell sorting (FACS) gating strategy for the isolation of Oct4-GFP+ cells from cultures undergoing reprogramming.

**Figure 3.**
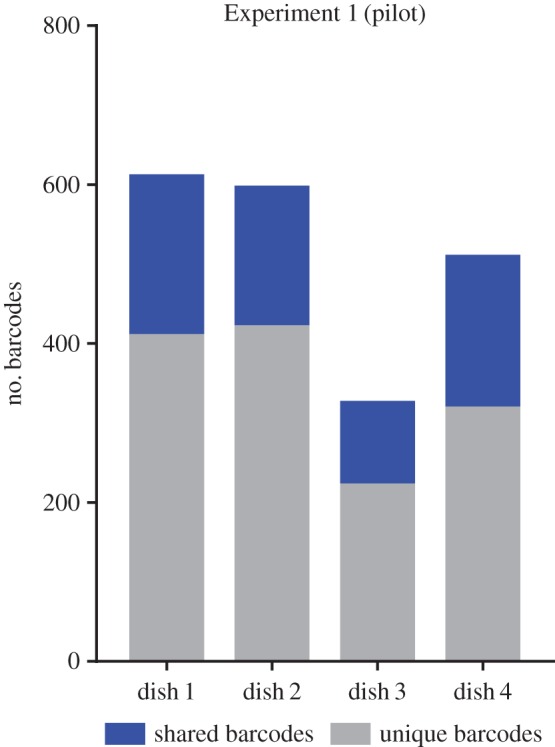
Pilot experiment; comparison of barcode representation in four dishes. The fraction of shared barcodes (marked as blue blocks) represents the barcodes observed in more than one dish. The number of unique barcodes specific for each dish is represented as grey blocks.

We first determined whether our reprogramming and barcoding systems function appropriately. We conducted a pilot experiment according to the above-mentioned experimental design (figure 1 and table 1). MEFs carrying Oct4-GFP transgene were cotransduced with lentiviruses encoding four factors and M2rtTA expressing barcoded lentiviruses. Infected cells were cultivated for 24 h (figure 2*a*) and then reseeded into four dishes on feeder cells. After this, we supplemented iPSC culture medium with DOX and AGi [20], thus initiating the reprogramming process. GFP-positive cells initially appeared on day 3 of culturing in this condition (figure 2*b*). By day 7, they formed both small and large colonies with round-shaped, tightly packed cells (figure 2*b*). Successfully reprogrammed cells were sorted for GFP (figure 2*c*), and DNA barcodes representing each dish were recovered (for more details, see Material and methods). Note that each unique recovered barcode corresponds to a colony of iPS cells. A significant proportion of barcodes was found to be shared between dishes (figure 3). We calculated the observed and expected number of shared barcodes; the latter case was based on the assumption of the stochastic nature of reprogramming supposing that clonally related cells were reprogrammed during the experimental timeline by a simple coincidence (see §4.10 Analysis of shared barcodes). According to our estimation, the observed number of shared barcodes (209) is much larger than expected (36), thus supporting the fact that some cell lineages within the heterogeneous cell population are more amenable to reprogramming and the potential to reprogramme is inherited by daughter cells during cell divisions.

**Table 1.** Summary of parameters for all experiments presented in this paper.

	Experiment 1	Experiment 2	Experiment 3	Experiment 4	Experiment 5
aim of experiment	pilot experiment: approbation of experiment's design	quantification of iPSC-forming lineages fraction among mixed population of MEFs	quantification of iPSC-forming lineages fraction among mixed population of MEFs	quantification of iPSC-forming lineages fraction among Thy+ population of MEFs	control experiment: random choosing of barcoded cells = simulation of stochastic reprogramming
the number of plated cells before transduction	170 000	230 000	200 000	190 000	190 000
the number of GFP-positive cells in the control experiments/MOI	59.1%/0.89	38.7%/0.49	29.1%/0.34	46.9%/0.63	46.9%/0.63
live imaging after transduction	No	50 h (electronic supplementary material, figure S2)	30 h (electronic supplementary material, figure S2)	28 h (electronic supplementary material, figure S2)	28 h (electronic supplementary material, figure S2)
computational modelling	No	yes (electronic supplementary material; figure 5*a*)	yes (electronic supplementary material; figure 5*b*)	yes (electronic supplementary material; figure 6*a*)	yes (electronic supplementary material, figure 6*b*)

We conclude that our system could be effectively employed for tracing cell lineages and analysis of their reprogramming potential. However, to quantify a fraction of these iPSC-forming lineages, we have to take into account some factors that confound lineage relationship assignment caused by cellular barcoding issues.

### 2.2. Analysis of reprogramming potential of clonally related cells considering cellular barcoding parameters confirmed symmetric reprogramming fate

The efficiency of the barcoding method to track clonal dynamics has been demonstrated in many studies [12,19,21–23]. The main principle underlying this method is based on introduction of an inheritable tag into the cellular genome. For effective cell labelling by barcodes, one has to consider the following factors. First is the library size or the library diversity; library diversity is a measure of the number of unique DNA barcodes that are available among barcode-bearing particles. Low library diversity leads to an accidental labelling of unrelated cells by the same barcode; thus, we risk assigning false lineage relationships and overestimating the number of barcodes shared between different dishes. To ensure single-cell representation, the barcode lentivirus library has to be large enough. Theoretically, barcode diversity is determined by the length of the random sequence (i.e. length of barcode). For our 30 bp barcodes, theoretical library diversity reaches 10^17^ unique variants. However, in practice, library diversity is restricted by various factors, such as inefficient barcode cloning into delivery vectors and production of viruses. We estimated library diversity by comparison of barcodes between several independent infections performed with the same barcoded virus batch (see §4.9 Barcoded library validation).

Another parameter is the number of barcode integrations into a single cell. The progenitor cells with multiple integrations of various barcodes will be interpreted as multiple progenitor cells with unique barcodes and the same fate, thereby increasing the number of shared barcodes between dishes. Reducing the probability of multiple barcoding is usually achieved by infecting cells with barcoded viruses at a low multiplicity of infection (MOI), and we followed this procedure. We wish to highlight that some cells will still be infected with more than one barcoded virus; however, knowing the MOI we can estimate their proportion.

Together, these parameters are essential for confidence in assigning lineage relationships during reprogramming. Full details of cellular barcoding methods with consideration of the challenges, power and limitations were exhaustively described in a review article by Naik *et al.* [24].

Besides the factors caused by the barcode library complexity, the number of progenies originating from the starting cells also influences the fraction of shared barcodes. Actually, during the population doubling time, a fraction of the cells divides more than once and another fraction dies in obscurity, for example. In general, the more progenies a given cell generates, the higher is their probability to be distributed in different dishes and fall under our scope. We used time-lapse microscopy to track starting cells during the pre-splitting period and estimated the fractions (%) of cells produced from 0 to several progenies (electronic supplementary material, figure S2; for more details, see Material and methods). Moreover, to account for PCR amplification and deep sequencing biases, we added a defined number of cells with known barcode sequences to each sample (for more details, see figure 1 and §4.6 Preparation of control cells).

**Figure 4.**
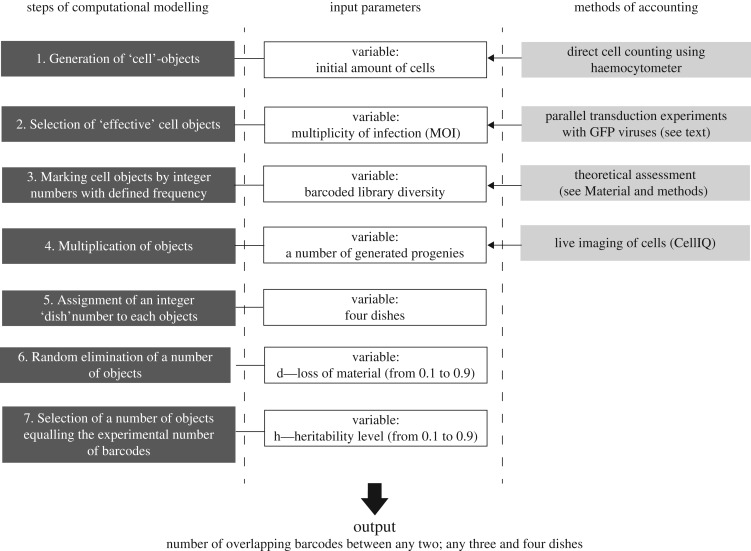
The outline of the computational model. The left column shows all steps of the proposed model. The central column shows input parameters that are used for performing simulation. The right column reflects the methods of accounting for the variables that vary from experiment to experiment.

To better understand how the potential to become an iPSC is distributed within a cell population, we developed a computational model mimicking the experimental procedure (see figure 4 and §4.11 Computational model and statistical analysis). Our model allows taking into account multiple experimental parameters such as the number of MEFs at the beginning of the experiment, the barcode library diversity, the MOI with barcoded viruses, the number of progenies generated from MEFs during population doubling time and the chance to plate these progenies into different dishes.

Moreover, the variation in control read counts between libraries from different dishes in one sequencing run highlighted the fact that PCR and sequencing introduce a bias (electronic supplementary material, figure S1*a*; for details, see §4.9 Barcoded library validation). It could also be seen there was a significantly different number of barcodes recovered from dishes in each experiment (electronic supplementary material, figure S1*b*) wherein there was no reason to believe that the reprogramming efficiency ranges dramatically among cells distributed in different dishes. To account for this, we introduced into our model an additional parameter—a loss of material. This parameter includes the loss of cells or barcodes in different stages of the experiment: from the not exactly equal division of cells into four dishes to variation in quality of PCR amplification and sequencing. It should be mentioned that we could not estimate the loss of material unambiguously, so in our model this parameter was varied from 10 to 90%.

Finally, the main parameter introduced into the model was a reprogramming heritability level, which reflects the probability of synchronous sister cell reprogramming. The output of the model is a number of shared barcodes: between any two dishes (double overlap), three dishes (triple overlap) and, finally, the number of barcodes presented in all dishes (quadruple overlap). The schematic of the computational model is shown in figure 4. The aim of the model was to define heritability level so that the simulated number of shared barcodes does not differ significantly from the number of shared barcodes observed in the reprogramming experiment. If several heritability levels satisfied this condition, we also selected a best-fit heritability level that was characterized by the median of the simulated number of shared barcodes closest to the average of shared barcodes observed in the reprogramming experiments.

**Figure 5.**
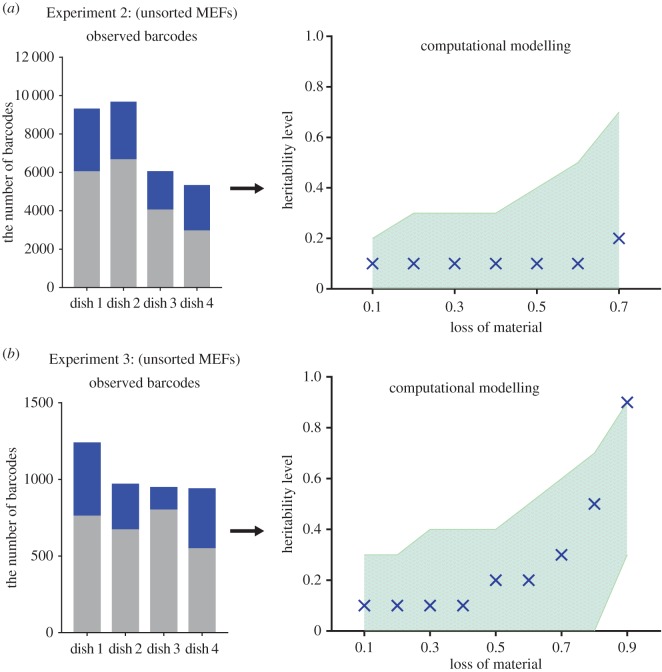
Clonally related cells often share the same reprogramming fate. Similar to figure 3, observed barcodes reflect the proportions of shared barcodes in each of the four dishes for the reprogramming Experiments 2 and 3 (*a* and *b*, respectively). Based on these experimental data, we performed a computational modelling to assess the level of synchronous sister cell reprogramming (i.e. ‘heritability’ level). The best-fit values of heritability plotted against the loss of material (in increments of 0.1 (10%)). Crosses indicate the best-fit values; shaded area indicates a range of heritability levels that satisfy the observed number of shared barcodes. All values of heritability were computed from a computational model mimicking the experimental data (figure 4). Simulation results were compared with experimental data using a non-parametric ANOVA test (Kruskal–Wallis test; *p* < 0.05). All statistical tests were performed using GraphPad Prism v. 7.00 software. The graphs showing the computational analysis of Experiment 2 do not include the values of heritability level at some points of the loss of material (points 0.8; 0.9) because the algorithm is not capable of modelling at these parameters.

We conducted two experiments considering the impact of the parameters discussed above (table 1). Again, we observed a significant number of barcodes shared between dishes, which indicates that the reprogramming might occur simultaneously in the many sister cells (figure 5*a,b*). This finding verified that the reprogramming potential is quite symmetric between sub-lineages originating from the same starting cell.

We simulated the experimental datasets using our computational model (figure 5*a,b*). The minimal ‘heritability’ level varies from 0 to 30% with a best-fit equalling 10% for both experiments. We noticed that with the increasing loss of material values the ‘heritability’ level increases as well. One should note that the number of simultaneously reprogrammed sister cells is directly limited by the total number of cells remaining after the loss of material. For example, in the extreme case when the number of remaining cells in each dish is equal to the size of observed overlaps, only 100% ‘heritability’ will satisfy the observed results. Thus, the larger loss of material values correspond to higher ‘heritability’ levels.

To circumvent the cellular heterogeneity in differential status and exclude rare somatic stem cells—the main contenders for the role of ‘elite’ subpopulation—we sorted ‘primary’ MEFs for fibroblast-associated marker Thy1 and conducted the reprogramming experiment according to the previously described scheme (Experiment 4; table 1). Simulation results of this dataset were consistent with those achieved previously. Unexpectedly, the minimal values of heritability were even more than those observed in the previous experiment, reaching at least 20% (figure 5*b*).

**Figure 6.**
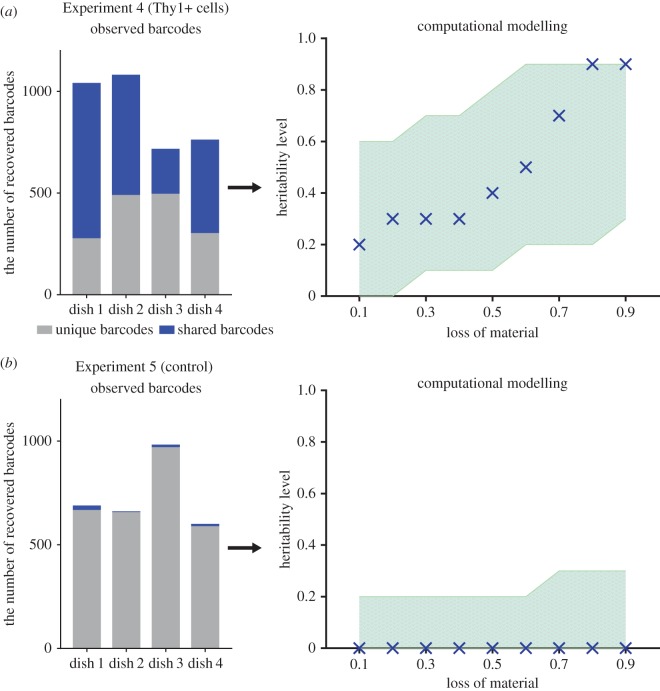
Probability of synchronous sister cell reprogramming significantly exceeds that caused simply by stochastic (random) events. Similar to figures 3 and 5, observed barcodes reflect the proportions of shared barcodes in each of the four dishes for the reprogramming of Thy1+ cells and the control experiment (*a* and *b*, respectively). The control experiment with randomly chosen barcodes (with the assumption that all barcodes have an equal chance of being selected) simulates the stochastic nature of reprogramming. Based on these experimental data, we performed a computational modelling to assess the level of synchronous sister cell reprogramming (i.e. ‘heritability’ level). The best-fit values of heritability plotted against the loss of material (in increments of 0.1 (10%)). Crosses indicate the best-fit values; shaded area indicates a range of heritability levels that satisfy the observed number of shared barcodes. All values of heritability were computed from a computational model mimicking the experimental data (figure 4). Simulation results were compared with experimental data using a non-parametric ANOVA test (Kruskal–Wallis test; *p* < 0.05). All statistical tests were performed using GraphPad Prism v. 7.00 software.

Furthermore, to make sure that the number of shared barcodes is not accidental (not due to the peculiarities of barcoding technique), we performed a control experiment with the same design, but instead of lentiviruses bearing reprogramming factors we used lentiviruses with DOX-inducible GFP reporter (Experiment 5; table 1). DNA-barcoding of these cells was performed under the same conditions as the reprogramming experiment. By *randomly* choosing cells from four dishes and analysing their barcodes, we could estimate the number of shared barcodes determined by *random* chance in a stochastic manner. Analysis of recovered barcodes showed a small proportion of shared barcodes in agreement with the fact that barcodes were chosen occasionally and there was no heritability at all (figure 6*a*). By simulating these parallel experimental datasets, we found that the ‘random heritability’ values caused only by chance were significantly lower than those in the reprogramming experiments (0% versus 20% for best-fit values). Together, these results support the privileged nature of particular cell lineages within the population and demonstrate that the exclusion of less differentiated cells from the Experimental cellular population does not affect the proportion of sister cells that reprogramme synchronously.

Finally, we estimated the contribution of cell subpopulations with different cycling properties to the overall number of double overlaps. We adapted our model to address this issue, based on the analysis of live-imaging data of dividing cells (electronic supplementary material, figure S2). We found that cells that produced more progenies (more than four in Experiments 2 and 3) account for more than 50% of the modelled overlaps (electronic supplementary material, figure S3), wherein their proportion in the total cell population does not exceed 25%. In this respect, the experiment for reprogramming of Thy1-positive cells differs from previous ones: cell division rate was shifted to the left with no one cell producing more than four progenies. It can be explained by the exclusion from the analysis of fast-cycling somatic stem cells. However, even in this case, the relative ratio of the contribution to double overlaps for cell-generated four progenies is larger than the ones for cell-generated two progenies.

Here, it is worth mentioning that our computational algorithm assigns an equal level of heritability and probability to be reprogrammed to all cells. An alternative possibility is represented by the situation when some cells, for example, fast-cycling cells, have a higher heritability level. There is no available information about the exact values of heritability levels for cells with different proliferative potential. Thus, we made several empirical selections of heritability levels and performed modelling. Sizes of double overlaps generated by the model using different heritability levels for fast- and slow-cycling cells were in agreement with observed data (electronic supplementary material, figure S4). Moreover, obtained triple and quadruple overlaps were even closer to the experimental results than in the model that uses a uniform distribution of heritability levels. Thus, it is possible that differences in proliferative potential of MEFs serve as a reason for increased reprogramming potential of defined cell lineages.

## 3. Discussion

In this study, we used the cellular barcoding tool for the simultaneous tracking of cell lineages contributing to iPSC generation. Our main findings show that clonally related sister cells often have equal reprogramming potential and generate pluripotent progenies with fixed latency. This may be explained by assuming that reprogramming potential is inherent to particular cell lineages and could be passed on through cell division. Most studies support a stochastic model of reprogramming, maintaining that activation of pluripotency markers occurs at different times in sister cells [10]. However, the hereditary component of the reprogramming potential cannot be entirely excluded because of the complexity of the study of the heterogeneous cell population and the difficulty of establishing cell relationships. Single-cell analysis of reprogramming events can overcome these limitations, but it is constrained by the relatively small number of analysed cells coupled with low reprogramming efficiencies, whereas by using cellular barcoding we could easily trace the fates of thousands of cells in a single experiment.

To more carefully estimate the distribution of the reprogramming potential within the cell population, we developed a computational model mimicking experimental conditions. According to the model, the minimal probability of synchronous reprogramming of sister cells is around 10–30%. If the reprogramming potential is acquired spontaneously in sister cells after the initial division (in a stochastic manner), the probability of synchronous reprogramming of cells within lineages will be determined only by the overall reprogramming efficiency. In our experiments, the efficiency did not exceed on average 4% (see §4.12 Calculation of reprogramming efficiencies), which is less than the minimal assessment of the reprogramming probability for sister cells (10–30%). This finding is also supported by the control experiment, which showed that if all starting cells had an equal chance of being selected, i.e. being reprogrammed, the probability to share ‘reprogramming fates’ would be significantly less. One of the possible explanations of these results is the existence of an ‘elite’ cell population that is privileged to reprogramming. It is well known that less differentiated somatic stem cells are more rapidly and efficiently reprogrammed to pluripotency [25]. We decided to exclude the ‘elite’ subpopulation of somatic stem cells from our analysis and repeated the experiment with MEFs sorted for the surface antigen Thy1, which is expressed at high level in mature fibroblasts. Interestingly, the proportion of synchronous reprogrammed cells remained the same, thus indicating that along with the less differentiated cells there might exist other subpopulations of cells predisposed to reprogramming. However, there exists one more possibility for the presence of a privileged cell population: only the cells harbouring specific viral integrations with a high level of reprogramming factors expression have a chance to be reprogrammed. Nevertheless, this explanation requires extreme differences between cells in the levels of OKSM expression, which is unlikely in the case of an expression driven by a similar strong promoter in all transduced cells.

Furthermore, we focused on the fate of fast-cycling cells during the reprogramming process. First, fast-cycling cells may have the same reprogramming potential as slow-cycling cells, which corresponds to our basic model with constant, uniform heritability level. Despite equal reprogramming potential, progenies of fast-cycling cells will be observed among reprogrammed cells more often only because of their number. It is important that our model simulates the different number of offspring for fast- and slow-cycling cells, and accounts for cells multiplying more rapidly having a higher chance to be split on different dishes. Still, the model suggests non-zero heritability levels, indicating that cells of the initial MEF population should have some inequalities in reprogramming potential in addition to the different cycling speed.

Our alternative model extends this hypothesis, suggesting fast-cycling cells as a subpopulation of MEFs characterized by a high reprogramming potential. In this model, characterized by non-uniform, proliferation-dependent heritability levels, both the high number of offspring and the high chance of each cell to be reprogrammed result in increased contribution of fast-cycling cells to the overall number of reprogrammed cells.

Models based on both uniform and non-uniform heritability levels satisfy the experimental results. Precise estimation of heritability levels in the non-uniform model is not possible because it requires adjustment of multiple variables (i.e. all heritability levels) based on a limited number of defined values (i.e. observed overlap sizes). However, a non-uniform model is preferable because it better explains the number of triple and quadruple overlaps. In this case, overrepresentation of fast-cycling cells is not only associated with a simple increase of cell numbers by divisions but is also due to certain intrinsic properties of these cells, for instance epigenetic predisposition to being reprogrammed. This finding is consistent with a recently published work by Pour *et al.* [26], which traced cell lineages from several divisions before factor induction and showed that predisposition to reprogramming is a heritable trait.

Thus, we suppose that reprogramming progresses in a partially deterministic manner at the level of cell lineages, at least. However, at the level of the total starting cell population, reprogramming is likely to be stochastic in accordance with the finding that the timing of faithful reprogramming varies widely among cells [10].

The road leading from differentiated cell to pluripotency passes through largely uncharted territory: iPS cells can be derived by various combinations of transcription factors and small molecules [27–29], and various components of the chromatin-modifying machinery affect reprogramming efficiency by accelerating or inhibiting induction of pluripotency [30,31]. The dynamics of reprogramming revealed that some cells transit to a pluripotent state along a short direct route and other cells choose the more entangled pathway requiring the maintenance expression of reprogramming factors for extended periods [8,10,32]. The choice of these routes is probably determined by pre-existing heterogeneities in the state of cells. According to our findings, some particular cells have a higher potential to reprogramme and could partially pass this on to progenies during division.

## 4. Material and methods

### 4.1. Viral constructs

To generate DOX-inducible lentiviral vectors, LeGO-G2 (Addgene #25917), a third-generation lentiviral vector, was first digested with *EcoRI* and *PspOMI* (to excise SFFV promoter and GFP gene) and further ligated with Tet operator, which was obtained from plasmid Tet-O-FUW-Ascl1 (Addgene #27150) using PCR amplification, cloned into T-vector (Promega) and finally digested with *EcoRI* and *PspOMI*. The resulting plasmid was named LeGO-TRE. The polycistronic cassette carrying all four factors linked with 2A peptides was excised from Tet-O-FUW-OSKM (Addgene #20321; courtesy of Dr. Kiselev) and subsequently cloned into the EcoRI site of LeGO-TRE. The resulting vector was named LeGO-TRE-OSKM. To create DOX-inducible GFP vector (LeGO-TRE-GFP), fragment coding GFP was excised from pLeGO-G2 using *BamHI* and *NheI* and then ligated into *BamHI* and *NheI* linearized LeGO-TRE.

To generate the lentiviral vector carrying transactivator M2rtTA (for expression of factors from TRE-promoters), eGFP was excised from LeGO-G2 with *EcoRI* and *BamHI* and replaced (using the same enzymes) by M2rtTA gene derived from FUW-M2rtTA (Addgene #20342) using PCR. The resulting vector was named LeGO-M2rtTA.

### 4.2. Construction of DNA barcode library

The barcode linker containing 30 random bases was created by annealing two oligos (forward, 5′-CATGTAACATGTCAGGAAACAGCTATGACC-3′; reverse, 5′-CATGTATGAATTCGTTGTAAAACGACGGCCAGTNNNNNNNNNNNNNNNNNNNNNNNNNNNNNNGGTCATAGCTGTTTCCTGACATGTTA-3′; Biosset) followed by extension by Phusion polymerase to create double-stranded barcode fragments. The left and right barcode flanking sequences contained EcoRI and PciI restriction sites. The barcode fragments were cloned into the non-expressing region of the LeGO-M2rtTA vector using EcoRI-PciI. The resulting vectors were transformed into (TOP10) competent *Escherichia coli* cells and grown on LB medium supplemented with ampicillin (50 g ml^−1^) for 2 h. Further, we plated 0.0015% of this transformation mix on an ampicillin-resistant plate and counted the number of colonies after overnight growth. The 727 colonies suggest that the complexity of the remaining library is approximately 48 500 000. From the rest of the transformation mix, plasmid libraries were harvested, purified with Plasmid Midiprep Kit (QIAGEN) and used for lentivirus particle production.

### 4.3. Lentiviruses production

Viruses were generated as described elsewhere [33]. Phoenix cells were transfected with a mixture of lentiviral vectors and packaging plasmids (pRVS-Rev, pMDLg/pRRE and pCMV-VSV-G) using Lipofectamine 2000 (Invitrogen). The medium was replaced 24 h after transfection, and viral supernatants were collected at 48 and 72 h postinfection. After filtration, supernatants were collected, aliquoted and stored at −80°C.

### 4.4. Cell culture and reprogramming experiments

Murine embryonic fibroblasts (MEFs) were derived from E13.5 embryos from transgenic OG mice carrying GFP under control of the Oct4 promoter [17] and expanded in fibroblast medium (DMEM with 10% FBS, l-glutamine, penicillin–streptomycin and non-essential amino acids). Time-pregnant mice were obtained from the Center for Genetic Resources of Laboratory Animals at the Institute of Cytology and Genetics, Siberian Branch, Russian Academy of Sciences (RFMEFI61914X0005 and RFMEFI61914X0010).

Cell counting and viability testing were performed using a haemocytometer. In each experiment, approximately 200 000 MEFs at passage two were infected overnight with two separate LeGO-based lentiviral vectors delivering LeGO-TRE-OSKM and LeGO-M2rtTA, respectively. It should be noted that infection procedures were performed with an excess of OSKM viral supernatant and a small amount of barcode-bearing M2rtTA viruses—for a low MOI level of barcoded viruses (to reduce multiple barcoding). MOI, the ratio of the number of virus particles to the number of target cells, was calculated using Poisson distribution from additional parallel experiments with the same parameters: equal numbers of MEFs was transduced with the same amount of barcoded viruses and an excess of viruses with DOX-inducible LeGO-TRE-GFP. The infection medium containing 5 µg ml^−1^ polybrene was replaced after 12–18 h with fibroblast medium and cells were incubated in CellIQ cell culturing platform (at the Microscopy Center of the Institute of Cytology and Genetics, SB RAS) for 24–48 h (until it reaches population doubling) with imaging. Images were taken within a several connected 3 × 3 spatial range at 20× magnification every 10 min and analysed manually. Further cells were reseeded into four dishes on feeder cells and cultured in ES medium (DMEM with 15% FMS, 15% Serum Replacement, l-glutamine, penicillin–streptomycin, non-essential amino acids, β-mercaptoethanol and 1000 U ml^−1^ LIF) supplemented with 2 µg ml^−1^ DOX and AG (GSK3β inhibitor (CHIR99021) 3 µM) and ascorbic acid (50 µg ml^−1^). Fresh ES medium with DOX was added every day during the experimental timeline (7 days), and then cultures were sorted for GFP by FACS. Testing for *Mycoplasma* was routinely performed. All cell cultures were maintained at 5% CO_2_ at 37°C.

### 4.5. Flow cytometry

For flow cytometry, cells were harvested by incubation in 0.25% trypsin/1 mM EDTA for 10 min at 37°C then resuspended in PBS and passed through a 40 µm cell strainer to achieve a single-cell suspension. Analysis and sorting of cells were conducted on a FACSAria instrument (BD Bioscience). For Thy sorting, MEFs at passage two were live-stained with anti-mouse CD90.2 (Thy1.2) antibodies conjugated to FITC (BioLegend Cat.# 140303). Cell suspensions at a density of 1 × 10^6^ cells/100 µl were incubated with 0.25 µg (at a 1/400 dilution) of the antibodies in MEF medium for 20 min at 4°C in the dark, then washed in PBS twice, harvested using trypsin/EDTA and resuspended in MEF medium. Then Thy1.2-positive cells were sorted as indicated. MEFs cultured without any manipulations were used as control.

### 4.6. Preparation of control cells

The Phoenix cells were infected with DOX-inducible GFP and barcoded LeGO-M2rtTA lentiviruses at low MOI such that no more than 10% of cells expressed GFP, in order to ensure that the most cells receive only one viral copy with high probability. Infected cells were expanded in culture with the addition of DOX (2 µg ml^−1^) and further GFP-positive cells were subcloned manually by single-cell isolation into a 24-well plate. These cells were cultivated for several weeks and some of them resulted in stable cell lines. PCR products from the genomic DNA of these cell lines were obtained using primers (forward, 5'-GTGGCCTGGAGAAACAGCTA-3'; reverse, 5'-CCACATAGCGTAAAAGGAGCA-3') then subcloned into pTZ57RT/T (Fermentas) and transformed into TOP10-competent *E. coli* cells. Sequencing analysis confirmed that each cell line contained a single unique barcode and two of them (TGCTGACCGCAGGTACGACGCCGAAGGATG—the first barcode; GGACGCGCCTACTACTTCGCGAGCATCCTG—the second barcode) were chosen as control. The defined number of these cells (100/20/10 cells for the first barcode and 400/80/50 cells for the second barcode) were combined with samples of cells sorted from each of the four dishes in Experiments 1–3, respectively (see §4.4 Cell culture and reprogramming experiments).

### 4.7. Barcode extraction and amplification

Genomic DNA was extracted from sorted GFP-positive cells with the use of the SDS/proteinase K method compatible with direct PCR amplification [34]. Cell pellets were resuspended in 5–10 µl of digestion buffer (0.005% SDS and 400 µg ml^−1^ proteinase K) and incubated for 3 h at 55°C. After heat inactivation of the proteinase K (for 10 min at 95°C), all volumes of digested samples were used for PCR amplification. During the PCR step, we used a high-fidelity DNA polymerase (New England Biolabs) and primers containing the adaptors necessary for Ion Torrent sequencing. Moreover, forward primers also contained 10 bp signature sequences that allow combining all four barcode libraries from the experiment (from four dishes) in one sequencing run with a 316 v2 chip. The PCR products at the correct size were extracted from 3% agar gel using PE Caliper LabChip XT with a DNA 300 chip. The assessment of NGS libraries quantity and molarity was performed with Agilent Bioanalyzer 2100. Purified PCR products were sequenced using the Ion Torrent sequencing platform (PGM) according to the manufacturer's instructions.

### 4.8. Next-generation sequencing data analysis

Sequencing data were processed using custom Python code. Since several indexed libraries were sequenced in one run, all raw sequences belonging to different libraries were sorted by unique 10 bp signature. The library sequencing qualities were checked using FastQC v. 0.10.1. The known sequences flanking DNA barcodes were removed using cutadapt 1.3. Moreover, low-quality sequences (20% of the bases with quality values less than 20) and sequences with length less than 26 bp and more than 34 bp were discarded as well. The remaining sequences were clustered using DNAclust tool according to the similarity of barcode sequences [35]. For more details regarding clustering parameters, see §4.9 Barcoded library validation. Barcodes with only one copy number were eliminated as a noise. Finally, barcodes shared between different dishes were counted using custom Python script. If the observed number of reads representing a barcode in a dish was less than 0.1% of the expected number (expected number of reads was calculated simply as a quarter of all reads containing the barcode), the barcode was not considered.

### 4.9. Barcoded library validation

The process of barcode recovery, namely PCR amplification and sequencing, may introduce mutations into the original barcode sequence. These mutated barcodes showing a high degree of sequence similarity with each other should be clustered together during data processing and should not be considered as independent. However, two independent barcodes presented in a library may accidentally show a high degree of similarity. Such barcodes should be considered as different and should not be clustered together. A parameter called identity threshold controls the balance between the two aforementioned scenarios during data analysis. We analysed how the value of identity threshold affects a number of independent barcodes identified in our data, as well as in simulated datasets containing 40 000 or 100 000 random 30 bp DNA barcodes. In both cases, the number of independent barcodes increased rapidly with the increase of identity threshold and reached a plateau at around 80% (electronic supplementary material, figure S6*a*). In the case of experimental datasets, the plateau was followed by a substantial increase of independent barcodes number when identity threshold was raised by over 90%. We interpreted this as an effect of background noise caused by PCR and sequencing errors. Thus, the optimal identity threshold for clustering, i.e. the threshold that makes it possible to ‘distinguish’ related barcodes despite the background noises, is 0.8 (80% similar sequence). This value was used to cluster barcodes using DNAclust tool.

To assess the library diversity, we compared DNA barcodes between a pair of three experimental datasets with barcoding of cells by lentiviruses from the overall viral batch. Barcodes shared in pairs reflect the occurrences of ‘repeat using’ barcodes; thereof the library diversity can be estimated. Estimated library size varies from experiment to experiment, ranging from 50 000 to 16 000 000 unique barcodes, with most estimations lying between 1 000 000 and 8 000 000. In addition, results of modelling were almost similar when library diversity varied from 50 000 to 8 000 000 (electronic supplementary material, figure S6*b*). We calculated library diversity for each experiment separately and used experiment specific values for modelling.

Moreover, we added a defined numbers of cells (varied from 10 to 400 cells for different experiments) containing known barcodes to each experimental library (each experiment included four libraries from four dishes) for the evaluation of PCR and sequencing quality. All control barcodes were detected and ranked in the right order of the number of cells from which these control sequences were obtained (electronic supplementary material, figure S1*a*). However, PCR introduces bias: it can be seen from the figure; in one sequencing run four libraries (from each dish) had a significantly different number of control reads while keeping correlation with an initial number of cells.

According to the literature, the number of tracked progenitors should be equivalent to 10% of the library diversity [24]. Based on the above analyses, we consider that our barcode library is well suited for effective labelling of a large number of cells (within half a million cells, at least).

### 4.10. Analysis of shared barcodes

We calculated the expected number of shared barcodes (*T*_expected_) using the formula6.1
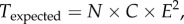
where *N* is the number of virus-transduced cells, *C* is the chance that two sister cells were split into different dishes and *E* is the efficiency of reprogramming. In our experiment, *N* was 170 000 cells, *C* was 0.75 (in the simple scenario, population doubling means that each cell generates two progenies with their probability of being split into four different dishes equalling 75%) and *E* was 00167 (for details, see §4.12 Calculation of the reprogramming efficiencies).

### 4.11. Computational model and statistical analysis

We designed a computational model that simulates all steps of our experiments (figure 4). The first step simulates the cell seeding and infection. Number (*N*_0_) of cells was calculated as6.2
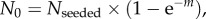
where *N*_seeded_ is the number of cells plated at the beginning of the experiment, and *m* is the MOI estimated for barcoded LeGO-M2rtTA lentivirus (figure 4, steps 1–2). Each cell was represented in a model as an independent object. Estimation of the amount of unique DNA barcodes (viral library diversity) was used to assign a barcode to each of the generated cells. Barcodes were represented by integer numbers, randomly selected from among 8 000 000 variants of unique barcodes (figure 4, step 3).

The next step simulates the period of cell cultivation in the absence of DOX (figure 4, step 4). Each of the generated cells was passed through a cell division procedure, which means that each cell gives rise to 0–8 progenies with the same barcode as those of the parental cell. The number of generated progenies was defined for each cell randomly according to the probabilities calculated during time-lapse experiments (electronic supplementary material, figure S2).

Next, generated cells were ‘plated’ on four separate dishes (figure 4, step 5). This was represented in the model as the assignment of a random integer number (named dish number) in diapason (1…4) to each cell object. Starting from this moment, all cells sharing the same dish number represent the appropriate culture dish. After ‘plating’, all dishes were subjected to a procedure named ‘cell death’, which means the random elimination of a defined number of cell objects from each dish (i.e. the loss of material; figure 4, step 6). The probability of cell illumination (d) varied from 0.1 (10%) to 0.9 (90%).

During the last (reprogramming) step (electronic supplementary material, figure S5, step 7), we reprogrammed a defined number of cells on each dish. In experiments, the number of different barcodes identified among reprogrammed cells varies from dish to dish. To account for this variance in the model, we calculated an average (*mu*) and variance (*si*) of observed barcode numbers and generate four random numbers BA_1_ … BA_4_ from a Gaussian distribution with the parameters *mu*, *si*. We reprogrammed a defined number of cells in each dish as presented in the electronic supplementary material, figure S5. This simulated the appearance of exactly BA_1_, BA_2_, BA_3_ and BA_4_ barcodes among reprogrammed cells on dishes 1, 2, 3, and 4, respectively (electronic supplementary material, figure S5).

Finally, all sisters of all ‘reprogrammed’ cells were selected, and each of them was defined as reprogrammed with a given probability (heritability level, h; electronic supplementary material, figure S5). The output of the model was the number of barcodes shared between different dishes. The number of shared barcodes was corrected, accounting the fact that for a given MOI a defined number of cells will harbour more than one integration of barcoded lentivirus (electronic supplementary material, figure S5).

The software was written in Python and executed on Novosibirsk State University High-Throughput Computing Cluster (http://www.nusc.ru/). We used at least 100 independent executions of the modelling algorithm for each given set of parameters. Simulation results were compared with experimental data using one-way non-parametric ANOVA test (Kruskal–Wallis test). All statistical tests were performed using GraphPad Prism v. 7.00 software.

### 4.12. Calculation of reprogramming efficiencies

Each barcode represents at least one pluripotent cell that has a reactivated Oct4 locus and has had the potential to form an iPS colony. Thus, we calculated the overall reprogramming efficiency by dividing the total number of recovered barcodes by the number of MEFs carrying barcoded virus. Because of low MOI (see barcoding aspects), only a fraction of seeded cells harboured barcoded viruses with LeGO-M2rtTA and hence had the chance of reprogramming. The ‘effective’ number of cells was determined from a given MOI as the percentage of cells infected with at least one viral particle using Poisson distribution. Calculated reprogramming efficiency varied from experiment to experiment with the median around 4.1%. It should be mentioned that some number of successfully reprogrammed cells remained undefined because of a lack of sequencing depth; thus our assessment of reprogramming efficiency based on the number of recovered barcodes is minimal.

## Supplementary Material

Supplementary material
