# Supplementary material for: Deterministic versus stochastic model of reprogramming: new evidence from cellular barcoding technique
Source: Open Biol. 2017 Apr 26;7(4):160311. doi: 10.1098/rsob.160311 (PMC5413903; doi:10.1098/rsob.160311)
Supplement: Supplementary material [file rsob160311supp1.docx]

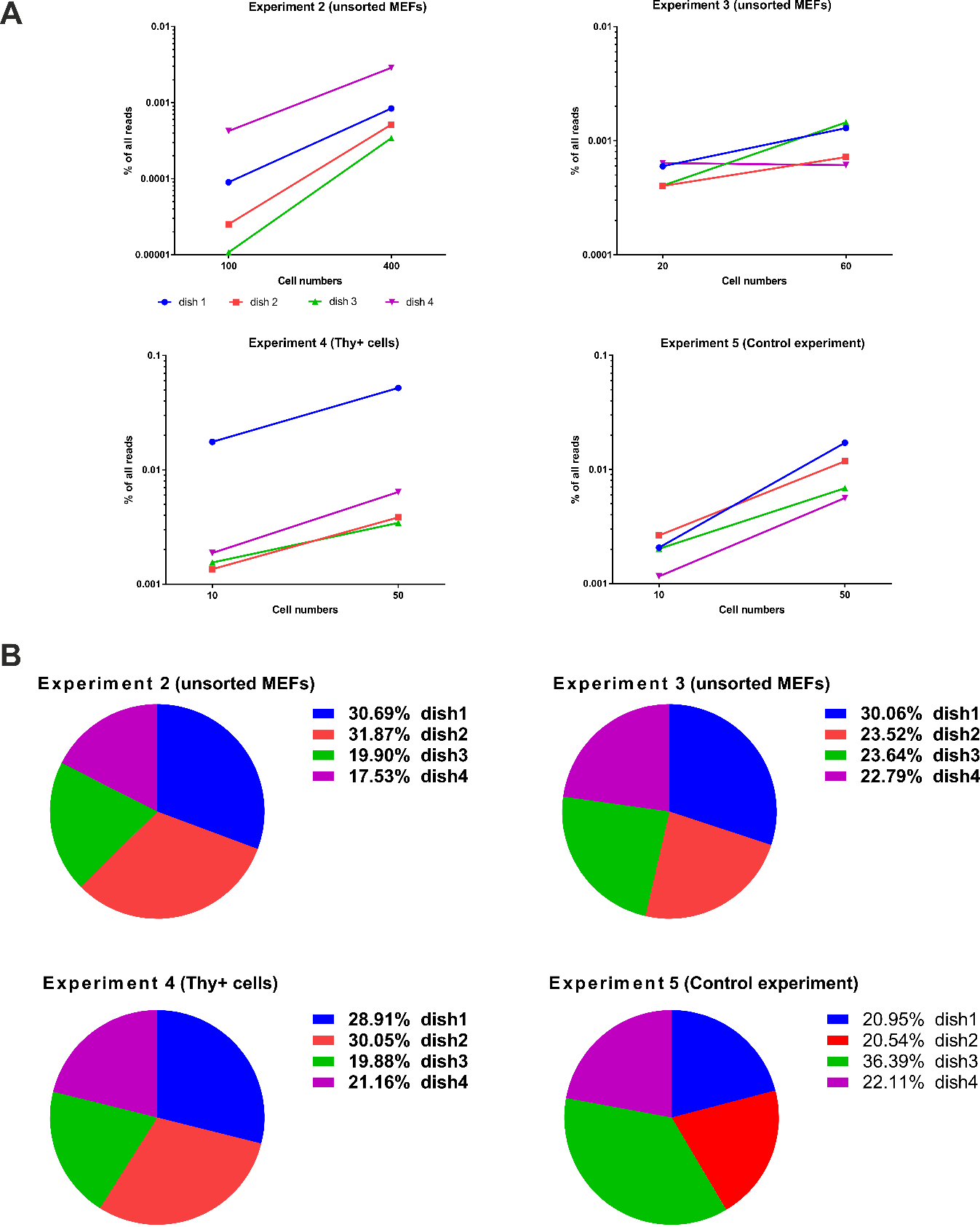


**Figure S1. The experimental procedures might introduce a bias.**

1. Relationships between absolute number of control cells and the fractional read value from the sequenced data (% of all reads). For details, see text and Materials and Methods.

1. Fraction of barcodes recovered from each of four experimental dishes in all reprogramming experiments.


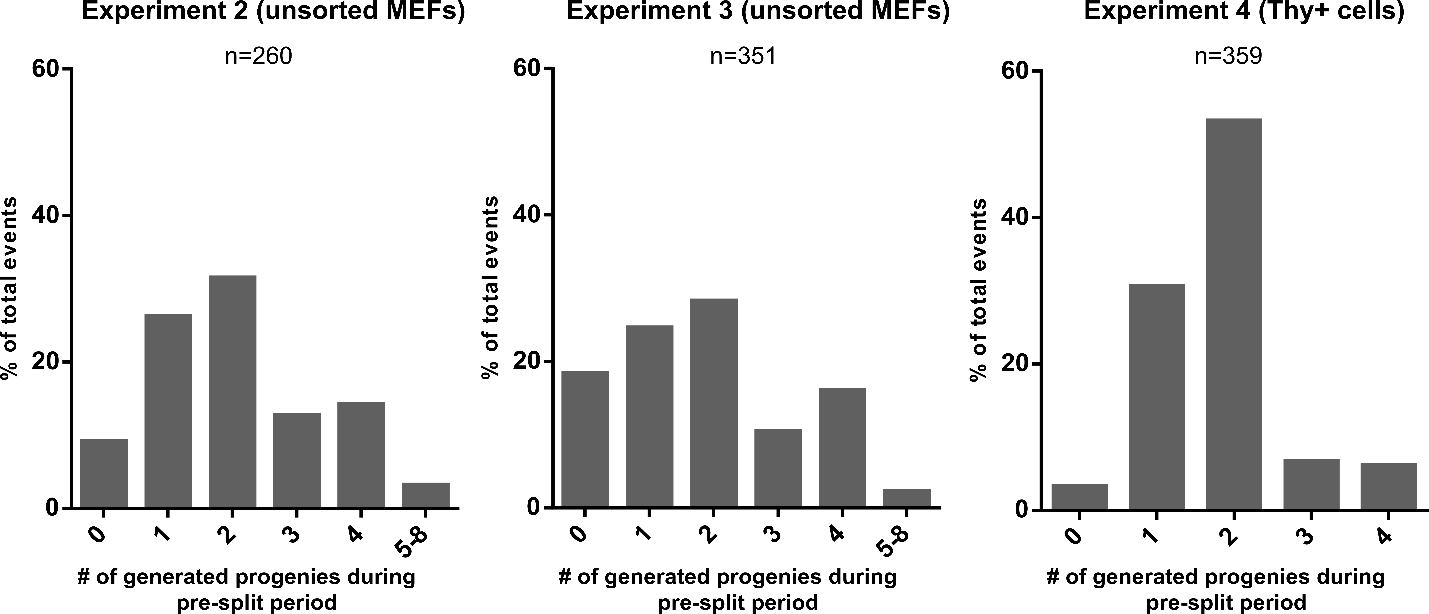


**Figure S2. Cell division tracking in transduced OG2 MEF during pre-split period (24-48 h).**

Histograms represent cell fractions that have died (0 progenies), have not divided (1 progeny), divided once (2 progenies), etc. All calculations were performed manually by analyzing the time-lapse images obtained with Cell-IQ.


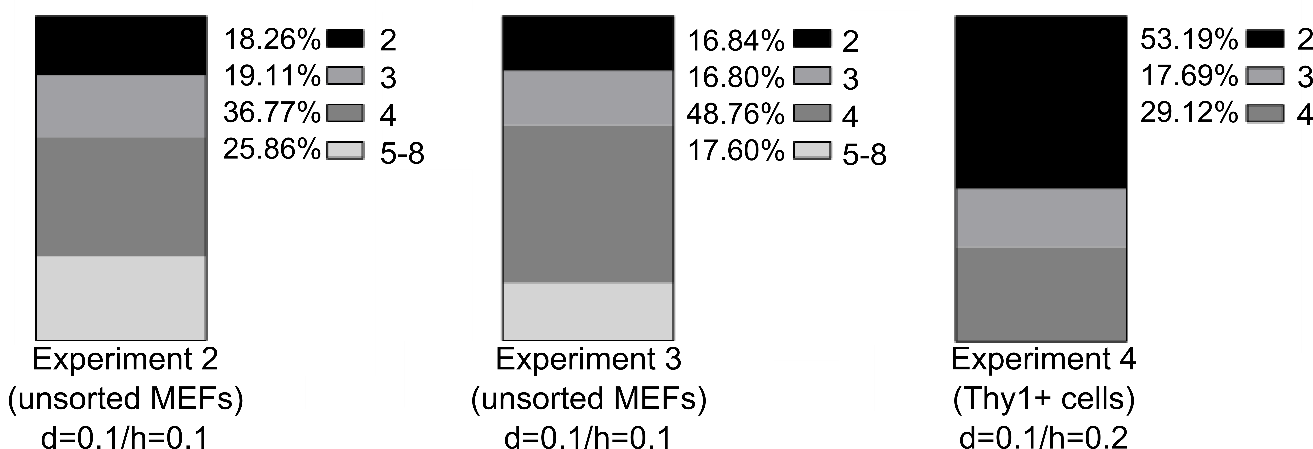


**Figure S3. Cells gave rise to more progenies contribute to reprogramming in a greater proportion.**

Histograms show a contribution of cell fractions with different number of produced progenies to overall amount of double overlaps. Data was obtained by computational analysis of double overlaps at the best-fit heritability value, h for the loss of material, d=0.1.


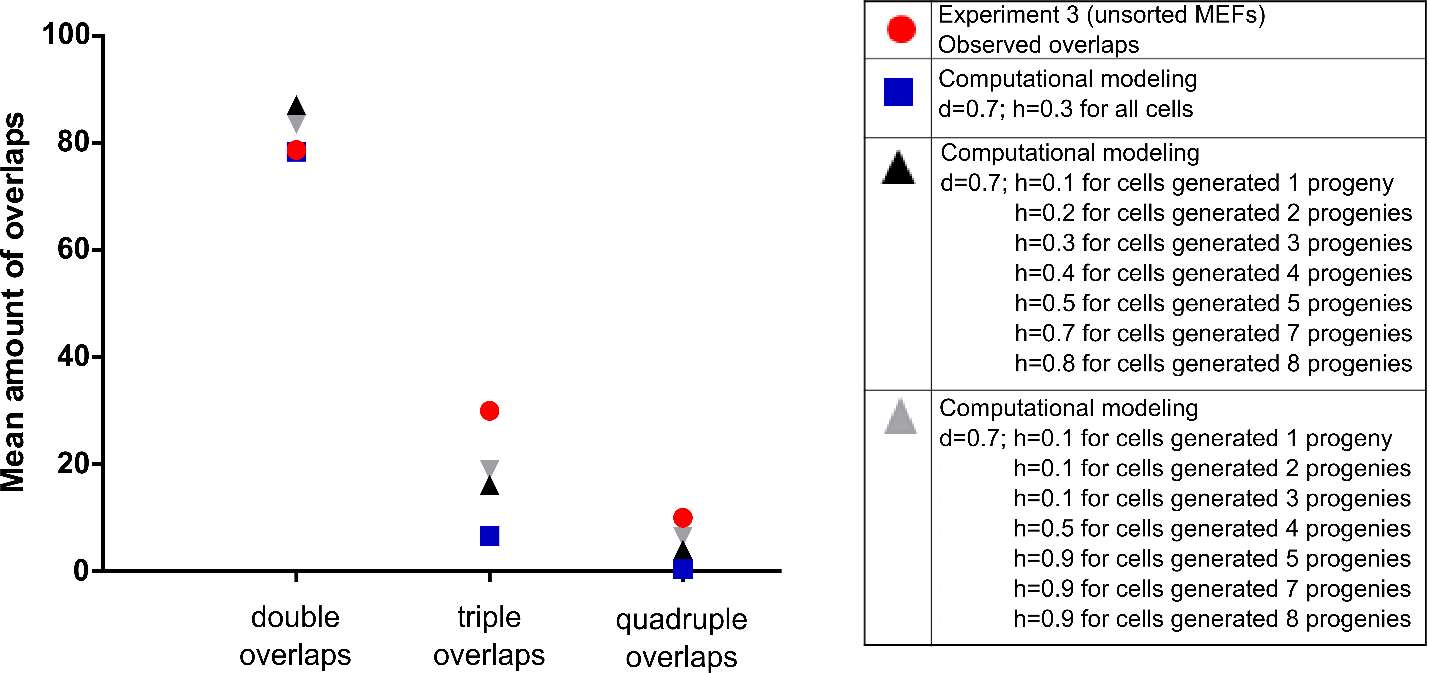


**Figure S4. Comparison of uniform and non-uniform heritability distribution models**

The graph represents the mean number of barcodes shared between dishes (for double, triple and quadruple overlaps) observed in the experiment 3 (red circles) compared to the model predictions (blue squares). The increasing similarity between observed and simulated overlaps achieving with increasing of heritability level for fast cycling cells (black and grey triangle). One of the best-fit values of the heritability level (d=0.7/h=0.2; where *d* means the loss of material and the *h* means the heritability level) was taken as the model parameters. The rest fitting values of heritability are not shown because they do not distinguish significantly from the results above.

**
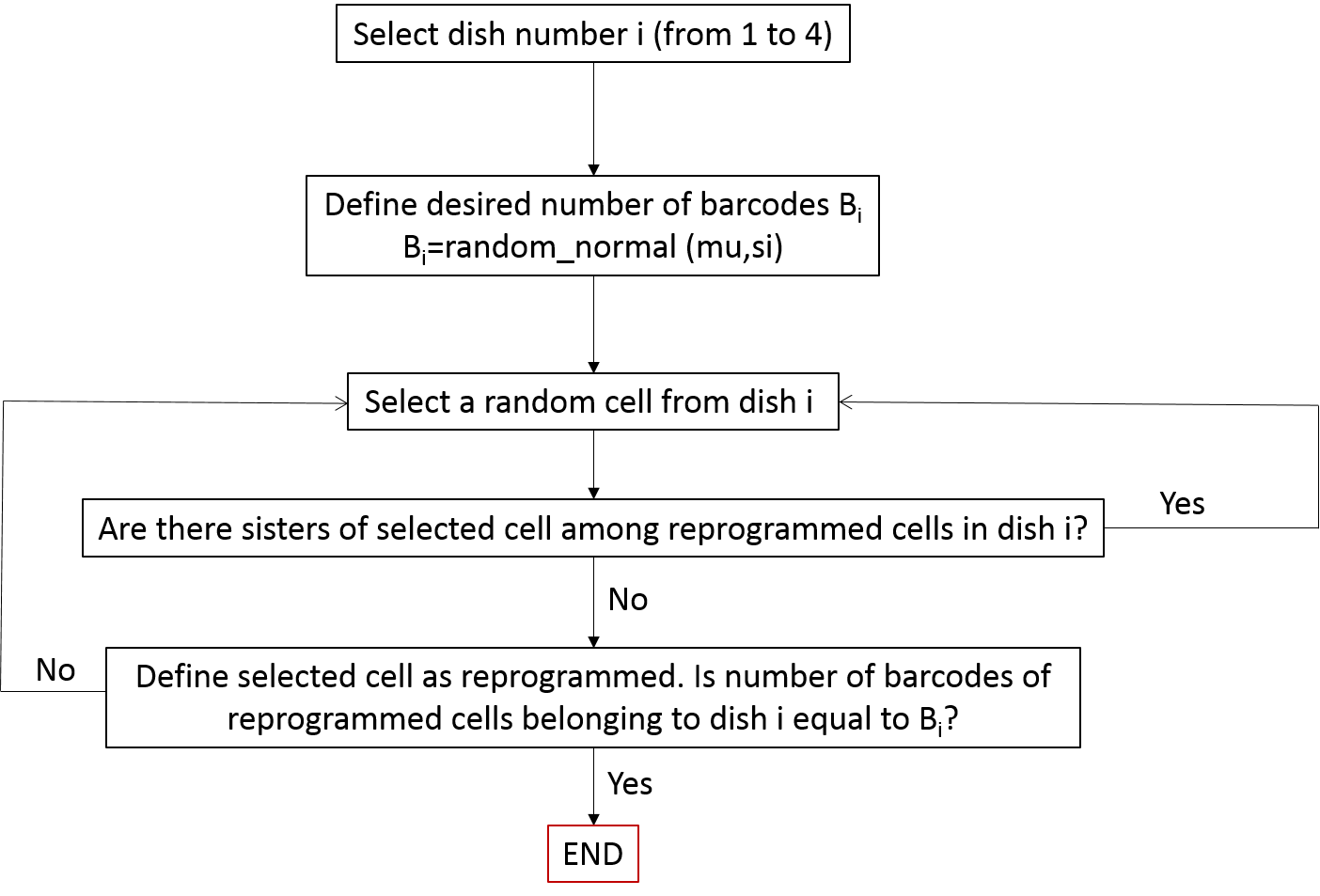
**

**Figure S5**. Schematic representation of model step 7 (reprogramming of cells).


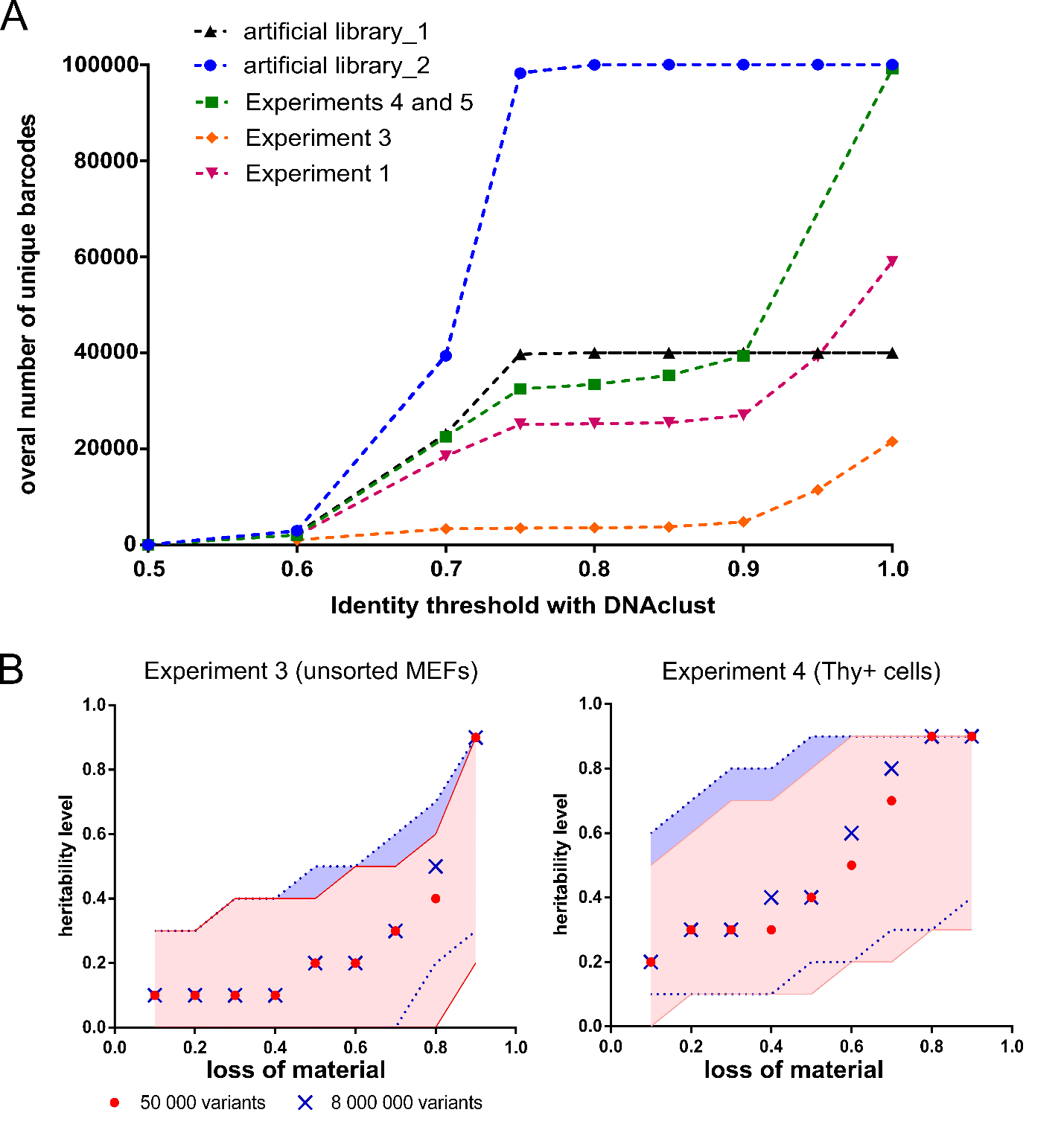


**Figure S6. Barcoded library validation**

1. Identification of appropriate identity thresholds. Real and simulated libraries clustered at various identity thresholds ranging from 100% down to 50% using DNAclust are shown. The artificial library_1 consists of 40 000 barcodes and the library_2 – of 100 000 barcodes.
2. Library diversity comparison. Results of modeling of Experiments 3 and 4 at different library sizes (50 000 and 8 000 000 unique barcodes). The best fit values of heritability i.e. the probability of reprogramming sister cells plotted against the loss of material (in increments of 0.1 (10%)). Crosses and dots indicate the best-fit values; shaded area indicates a range of heritability levels that satisfy the observed number of shared barcodes. All values of heritability were computed from a computational model mimicking the experimental data. Simulation results were compared with experimental data using a nonparametric ANOVA test (Kruskal – Wallis test; p < 0.05). All statistical tests were performed using GraphPad Prism 7.00 software.
